# Should ICSI be implemented during IVF to all advanced-age patients with non-male factor subfertility?

**DOI:** 10.1186/s12958-019-0474-y

**Published:** 2019-03-07

**Authors:** Jacob Farhi, Kfir Cohen, Yossi Mizrachi, Ariel Weissman, Arieh Raziel, Raoul Orvieto

**Affiliations:** 1 IVF Unit, Wolfson Medical Center, Holon, Israel; 2 Sackler Faculty of Medicine, Tel Aviv University, Tel Aviv, Israel; 3 Infertility and IVF Unit, Department of Obstetrics and Gynecology, Chaim Sheba Medical Center (Tel Hashomer), Ramat Gan, Israel; 4 Tarnesby-Tarnowski Chair for Family Planning and Fertility Regulation, Sackler Faculty of Medicine, Tel-Aviv University, Tel Aviv, Israel

**Keywords:** ICSI, IVF, Insemination, Maternal age, Fertilization rate, Top-quality embryo

## Abstract

**Backgroud:**

In order to maximize In vitro fertilization (IVF) success rates in advanced- age patients, it has been suggested to favor the use of intracytoplasmic sperm injection (ICSI) over conventional insemination (CI), with the notion that ICSI would serve as a tool to overcome interference in sperm oocyte interaction and sperm oocyte penetration issues that can be related to maternal age and are not due to sperm abnormalities. We therefore aim to evaluate the role of ICSI in the treatment of non-male factor infertile patients aged ≥35 in terms of fertilization and top-quality embryo rates.

**Methods:**

In this retrospective cohort study, data were collected and analyzed for all patients with non-male factor infertility, aged ≥35 treated, undergoing their first IVF cycle attempt with 6 or more oocytes yield, in whom a 50% ICSI-CI division was performed.

**Results:**

Five hundreds and four oocytes were collected from 52 eligible patients. Overall, 245 oocytes underwent ICSI and 259 oocytes underwent CI. The fertilization rate was 71.0% following ICSI, compared to 50.1% in the CI treated oocytes (*P* < 0.001). The top quality embryo rate was 62.8% following ICSI compared to 45.5% following CI (*P* < 0.001). Subdividing the study population to two age groups revealed that the above differences remained significant in patients aged 35–39 yrs., whereas in those aged 40–45 yrs., the differences were non-significant but still inclined in favor of ICSI.

**Conclusions:**

This study favors the use of ICSI in the older IVF population in order to increase both the fertilization rate and the number of top quality embryos that result per IVF cycle. Further studies are needed to establish our observations and use ICSI as the preferred approach to overcome egg sperm abnormal interactions related to advanced maternal age.

## Introduction

Intracytoplasmic sperm injection (ICSI) was developed for the treatment of couples with severe male-factor infertility to enable fertilization and pregnancy rates regardless of semen characteristics. Since its introduction [1], ICSI has gained tremendous popularity in assisted reproductive technology (ART) units throughout the world [1, 2] and has became a routine procedure in many of them. A recent report published by the Centers for Disease Control assessing data from the majority of IVF clinics in the USA has reported significantly different ICSI utilization and live birth rates per cycle, across geographical regions of the U.S. Moreover, higher ICSI utilization rate (up to 93.5%) was not associated with higher rates of male factor infertility nor were they strongly correlated with higher live birth rates per cycle [3].

In addition to severe male factor infertility, ICSI is currently being applied in patients with fertilization failure or low fertilization in previous standard in vitro fertilization (IVF) procedures. Morton et al. [4] reported that ICSI rescue attempts at 20–24 h after primary failure of IVF cycles resulted in normally fertilized oocytes but a low pregnancy rate. Alternatively, while Kastrop et al. [5] found that ICSI yielded better results than IVF with high insemination concentrations or microdrops in patients with previous failed fertilization, this observation was not substantiated by Tournaye et al. [6] who reported no difference in terms of fertilization between the two methods.

Thus, although ICSI provides a powerful tool to overcome previous reduced or total failure of fertilization in couples with unexplained infertility, its benefit for other types of infertility remains questionable. Likewise, opinions are divided regarding the notion that ICSI will be used as the procedure of choice in all couples requiring assisted reproduction techniques in order to prevent up to 30% rate of fertilization failures in the first conventional IVF attempt [6].

Age alone has a detrimental impact on fertility, mostly due to the significant increase in aneuploidy and spontaneous abortion rates with advanced maternal age [7]. Moreover, advanced maternal age is a known cause for oocyte quality degradation and poor IVF outcome. In order to maximize the success rates in terms of fertilization and top-quality embryo rates in advanced-age patients, it has been suggested to favor the use of ICSI over conventional insemination (CI) with the notion that ICSI would serve as a tool to overcome interference in sperm oocyte interaction and sperm oocyte penetration issues [8] that can be related to maternal age and are not due to sperm abnormalities. However, a recently published retrospective study including 745 women did not show an advantage for ICSI over conventional IVF in women above 40 years of age with non-male factor infertility [9].

In 2004, the Cochrane Group dealt with the issue of whether ICSI improves live-birth rate in comparison with CI in couples with non-male factor subfertility [10]. It was concluded that since no randomized data comparing live-birth rates exists, further research are required to elucidate whether ICSI should be preferred to IVF for cases of non-male factor subfertility. Prompted by the aforementioned information, we elect to evaluate the role of ICSI in the treatment of non-male factor infertile patients aged ≥35, with respect to fertilization and embryo quality rates as primary endpoints.

## Patients and methods

We reviewed the computerized files of all consecutive women admitted to our IVF unit during 10-years period, and reached the ovum pick-up (OPU) stage. The elimination of bias in this selection, for the purposes of this study, was achieved by including only patients’ aged ≥35, undergoing their first IVF cycle attempt for non-male factor infertility [11]. The presence of both ovaries and a uterine cavity with no abnormalities that might impair endometrial receptivity or embryo implantation were required. The study was approved by our institutional review board.

All the accepted protocols for ovarian stimulations were included. The selection of type of controlled ovarian hyperstimulation (COH) protocol used was the decision of the treating physician and largely dependent on the fashion at the time. The oocytes retrieved from each patient were divided into two groups, those that underwent CI and those undergoing ICSI. Fertilization was confirmed by observation of two pronuclei 18 to 19 h after IVF insemination or ICSI. Total fertilization rate (per group) was calculated as the total number of zygotes divided by the total number of oocytes. Embryos classification was based on the individual embryo scoring parameters according to pre-established definitions [12]. While a top quality embryo (TQE) was defined as two to four, or six to eight blastomeres on day 2 or 3 respectively, with equally-sized blastomeres and < 15% fragmentation, poor quality embryos consist of all the rest.

Data were collected and analyzed for all patients aged ≥35 treated for non-male factor infertility in whom 6 or more oocytes were retrieved and a 50% ICSI-CI division was performed in their first IVF cycle attempt. While the ICSI\CI division policy was applied for all non-male factor infertility patients undergoing their first IVF cycle, we selected to include only those achieving a minimum of 6 oocyte, in order to enable a feasible sibling oocyte comparison.

Results are presented as means + standard deviations. Differences in variables were statistically analyzed by Wilcoxon and chi-square tests, as appropriate. A *p* value of less than 0.05 was considered significant.

## Results

Fifty-two patients undergoing their first IVF cycle attempt for non-male factor infertility met the inclusion criteria and were evaluated in our analysis. Mean patients’ age during the study period was 38.7 + 2.6 (range 35–45) years. Causes of infertility were: unexplained in 33 (63.5%) patients, tubal factor in 17 (32.7%) and 2 patients (3.8%) with endometriosis. Thirty-four patients underwent COH using the multiple-dose GnRH-antagonist protocol and 18 underwent the long GnRH-agonist suppressive protocol. Both protocols resulted in comparable stimulation variables; e.g. similar peak estradiol levels (2328 + 1079 vs 2437 + 1197. *P* = 0.57, respectively) oocytes yield (9.6 + 3.7 vs 9.9 + 2.4, *p* = 0.8, respectively), with a significantly higher fertilization rate while using the long GnRH-agonist suppressive protocol (5.2 + 2.9 vs 7.0 + 2.8, *p* < 0.03).

Following the 52 IVF cycles, 504 oocytes were collected. The mean number of oocytes retrieved per cycle was 9 (range: 6–12). Overall, 245 oocytes underwent ICSI and 259 oocytes underwent CI. The median numbers of oocytes undergoing ICSI and CI were 4 (ranges: 3–9 and 3–14, respectively).

The fertilization rate was 71.0% (174/245) following ICSI treated oocytes compared to 50.1% (130/259) in the CI treated oocytes (*P* < 0.001). Moreover, top quality embryo rate was 62.8% (154/245) following ICSI compared to 45.5% (118/259) following CI (P < 0.001). These significant differences were also maintained when comparing the mean fertilization (0.69 ± 0.27 vs 0.54 ± 0.33, *p* < 0.005, respectively) and top- quality embryos (0.66 ± 0.50 vs 0.51 ± 0.47, *p* < 0.05, respectively) per patient.

Patients were further divided into two sub-groups according to their age, 35–39 (*n* = 31) and 40-45 yrs. (*n* = 21). In the 35–39 age sub-group, the fertilization and top quality rates were significantly higher in the ICSI treated oocytes compared to CI. Fertilization rate was (111/151) 73.5% vs (83/151) 54.9% (*p* < 0.001) and the top quality embryo rate was (93/151) 61.5% vs (75/151) 49.6% (p < 0.001) in the ICSI vs. CI oocytes, respectively.

In the 40–45 age sub-group, the fertilization rate [(63/94) 67.0% vs. (47/108) 43.5%; *P* < 0.057] and the top quality embryo rate [(61/94) 64.8% vs. (43/108) 39.8%; P < 0.057] were non-significantly higher in the ICSI vs. CI oocytes, respectively. Although result did not reach statistical significance, the numerical difference between ICSI and CI is still striking and strongly suggest of the priority of ICSI over CI in this age group.

Since the decision on which embryo should be selected for transfer, was based solely on embryo morphology and not whether, it was originated by ICSI or CI, interpretation of our data with respect to implantation, clinical pregnancy or live birth rates was limited. However, we still find it important to present IVF results for the entire IVF study population. Regarding the all study group, the implantation, clinical pregnancy, miscarriage and live birth rates were 13.9% (40/286), 30% (16/52), 11.5% (6/52) and 19% (10/52), respectively. Seven pregnancies resulted in singleton and 3 with twins’ deliveries. Since embryo transfers also consisted of embryos originated from both ICSI and CI, we could not assess the effect of ICSI vs CI on implantation or live birth rates.

In 18 patients embryos originated only for CI or ICSI were transferred. Although it is a very limited comparison, we present the results without interpretation. Of the six patients who underwent a transfer of embryos following CI, 2 (33%) conceived, compared to 8 (66%) out of the 12 patients undergoing transfer of embryos solely following ICSI.

## Discussion

In the present cohort-historical study, patients aged ≥35 years undergoing their first IVF cycle attempt for non-male factor infertility, yielded significantly higher fertilization and top-quality embryo rates, while applying ICSI vs CI on their sibling oocytes. Sub-dividing the study population by age revealed that these differences remained significant in patients aged 35–39 yrs. Although for those aged 40–45 yrs. the differences were non-significant, the results are still indicative that ICSI is beneficial.

Researching the literature with regards to the comparison of ICSI vs CI in patients with non-male factor infertility reveals controversial results. Aboulghar et al. [13] prospectively studied patients with borderline semen characteristics or unexplained infertility who underwent conventional IVF and ICSI on sibling oocytes. In the 24 patients with borderline semen characteristics, the ICSI procedure added a clear advantage in fertilization rate (59% vs 27.1% for IVF) and in cases of total fertilization failure (0 vs 45.8%). However, in the 22 patients with unexplained infertility, ICSI yielded better results than IVF only in those with total fertilization failure (0 vs 22.7%, respectively). The authors concluded that performing ICSI and conventional IVF for sibling oocytes in these groups of patients may spare 34.8% of them from embryo transfer cancellation due to total fertilization failure with conventional IVF. These observations are in accordance with those of Ruiz et al. [14], who studied couples with unexplained infertility or mild endometriosis undergoing IVF after four failed IUI cycles. Sibling oocytes were randomized into IVF or ICSI insemination groups. Although no significant between-group difference was observed in fertilization rate, there were no cases of total fertilization failure in the ICSI group, compared to an 11.4% total fertilization failure rate in the IVF group. Similarly, Staessen et al. [15] found comparable fertilization rates, cleavage rates, embryo quality and embryo implantation potential for ICSI and conventional IVF in sibling oocytes from couples with tubal infertility and normozoospermic semen. However, complete fertilization failure occurred in 12.5% of the oocytes undergoing conventional IVF compared to 3.6% of the oocytes undergoing ICSI. Khamsi et al. [16] reported total fertilization failure rates of 14.3% in the IVF group compared to only 2.9% in the ICSI group. Yang et al. [17] studied sibling oocytes from women in whom male factor was not involved. A similar fertilization rate was achieved in the ICSI and IVF groups, but embryo quality and implantation rate were higher after ICSI. Oehninger et al. [18] also reported higher quality embryos after ICSI in couples with teratospermia, whereas, Ruiz et al. [14] failed to note any such difference. The superiority of ICSI embryos may be explained by the fact that the ICSI procedure avoids oocyte and zygote culture with a lot of spermatozoa. This thereby reduces exposure to the reactive oxygen species produced by the spermatozoa that might contribute to embryonic damage [19].

In contrary to the aforementioned studies, in our study we included only patients > 35 years, and demonstrated improved IVF cycle outcome using ICSI vs CI. Our observations are in contrary to those recently published by Tannus et al. (9). In their retrospective study including 745 women, they could not demonstrate any advantage for ICSI over CI in women above 40 years of age with non-male factor infertility. Although similar numbers of oocytes were retrieved in both groups, the IVF group had a higher numbers of MII oocytes, higher fertilization rates per oocyte retrieved and higher numbers of zygotes formed. A potential explanation for this discrepancy may result from the possibility that oocytes might have been scrutinized for maturity upon ICSI and immature oocytes were discarded. While in CI, the maturity of oocytes is not examined until 16–18 h after insemination and the cumulus–oocyte complex is maintained intact in culture allowing more oocytes to complete in-vitro maturation and subsequently fertilize.

The present study was limited by its retrospective design. Moreover, one may argue that while dividing the oocytes to ICSI and CI, the embryologist may select the more “mature” oocytes for ICSI, thus, skewing the results in favor of ICSI. Moreover, due to the small sample size, we could not relate the fertilization methods to pregnancy and live birth rates. However, the results of our study strongly favour ICSI in attempting to overcome age related abnormalities in sperm egg interaction. Moreover, there is enough support for this approach in other studies presented above to encourage more research on this topic.

## In conclusion

Patients with unexplained infertility form a heterogeneous group. In those with undetected sperm abnormalities, ICSI may provide an advantage over CI. Although it does not increase the fertilization rate, it does avoid the devastating complete fertilization failure of the CI cycle, which takes an enormous physical, emotional and financial toll on affected couples.

In advanced-age patients with non-male factor infertility, specifically in the 35–39 female age group, the use of ICSI should be considered in most (if not all) oocytes in the first IVF attempt in order to maximize fertilization and top-quality embryo rates. Further large prospective studies are needed to elucidate the aforementioned recommendation and prior to its routine implementation.

## Abbreviations

ART: Assisted reproductive technology
COH: Controlled ovarian hyperstimulation
ICSI: Intracytoplasmic sperm injection
IVF: in vitro fertilization
OPU: ovum pick-up
TQE: top-quality embryo
